# Low-Cost Tetraplex PCR for the Global Spreading Multi-Drug Resistant Fungus, *Candida auris* and Its Phylogenetic Relatives

**DOI:** 10.3389/fmicb.2018.01119

**Published:** 2018-05-29

**Authors:** Amir Arastehfar, Wenjie Fang, Hamid Badali, Afsane Vaezi, Weiwei Jiang, Wanqing Liao, Weihua Pan, Ferry Hagen, Teun Boekhout

**Affiliations:** ^1^Westerdijk Fungal Biodiversity Institute, Utrecht, Netherlands; ^2^Department of Dermatology, Shanghai Changzheng Hospital, Second Military Medical University, Shanghai, China; ^3^Shanghai Key Laboratory of Molecular Medical Mycology, Shanghai Institute of Medical Mycology, Shanghai Changzheng Hospital, Second Military Medical University, Shanghai, China; ^4^Department of Medical Mycology/Invasive Fungi Research Center, School of Medicine, Mazandaran University of Medical Sciences, Sari, Iran; ^5^Student Research Committee, Mazandaran University of Medical Sciences, Sari, Iran; ^6^Institute for Biodiversity and Ecosystem Dynamics, University of Amsterdam, Amsterdam, Netherlands

**Keywords:** end-point PCR, *Candida auris*, multi-drug resistance, animal model, clinical validation, molecular diagnosis

## Abstract

*Candida auris*, *C. haemulonii*, *C. duobushaemulonii*, and *C. pseudohaemulonii* are closely related and highly multidrug resistant yeast pathogens. The high cost and low accuracy of current diagnostics may underestimate their prevalence, especially in medical resource-limited regions. In this study, we used 172 *C. auris* stains and its relatives and 192 other fungal strains to establish and validate a novel multiplex end-point PCR. A prospective and a retrospective clinical screenings using this assay were further performed in China and Iran respectively. We identified the first isolate of *C. pseudohaemulonii* in China and the first isolate of *C. haemulonii* in Iran from 821 clinical isolates in total, without any false positive. Animal models of *C. auris* and *C. haemulonii* were established for validation. The overall positive rates of the assay for mice blood and tissue were 28.6 and 92.9%, respectively. Compared with previously developed assays, our assay is more available and affordable to the developing countries, and may contribute to a better understanding of the epidemiology of *C. auris* and its relatives in these regions.

## Introduction

The evolving epidemiology of non-*albicans* *Candida* species and other emerging opportunistic pathogenic yeasts contributes to an increasing morbidity and mortality globally (Miceli et al., 2011; Fang et al., 2017). Among them, *Candida auris* and its medically important relatives in the *Metschnikowiaceae* clade, e.g*., C. haemulonii*, *C. duobushaemulonii*, and *C. pseudohaemulonii*, are well known as highly multidrug resistant pathogens that can lead to both superficial and deep-seated infections (Cendejas-Bueno et al., 2012). *C. auris* appears to be the most notable species at present due to its outbreak potential and high mortality rate (30–50%) (Calvo et al., 2016; de Almeida et al., 2016). After the first isolate from the external ear canal of a Japanese inpatient in 2009, a number of severe *C. auris* cases occurred subsequently in 17 countries from five continents (Chowdhary et al., 2017; Mohsin et al., 2017; Arauz et al., 2018). Meanwhile, other phylogenetic related species (*C. haemulonii*, *C. duobushaemulonii*, and *C. pseudohaemulonii*) share similar characteristics of *C. auris* and are also emerging worldwide (Sugita et al., 2006; Ramos et al., 2015; Boatto et al., 2016; de Almeida et al., 2016; Fang et al., 2016; Kumar et al., 2016).

Besides the antifungal profile and outbreak characteristics, clinical intervention for *C. auris*, *C. haemulonii*, *C. duobushaemulonii*, and *C. pseudohaemulonii* infections are mainly challenged by diagnostic difficulties. Firstly, the most commonly used identification systems, e.g., VITEK and API-20C AUX, are time-consuming and tend to misidentify *C. auris* as other yeasts, such as *C. haemulonii*, *C. sake*, *C. famata*, *Rhodotorula glutinis*, *Saccharomyces cerevisiae* (Chowdhary et al., 2017). Secondly, more specific diagnostic methods (e.g., sequence-, RT-PCR- and MALDI-TOF MS- based) require high-cost equipment and trained technicians (Girard et al., 2016; Kordalewska et al., 2017). Thirdly, although there are some in-house developed detective systems, such as the updated MALDI-TOF MS library for *C. auris*, there is no FDA approved one that can be used in the clinic till now (Mizusawa et al., 2017).

The high cost, low accuracy and unavailability of current diagnostics tools may underestimate the global prevalence of these pathogens, especially in medically resource-limited regions (such as the Africa and Southeast Asia). Consequently, this may lead to the fact that nearly all the reported cases were from developed countries (such as United States, United Kingdom, and Germany) or medically developed cities in the low-income regions (such as New Delhi, India) (Kumar et al., 2016; Chowdhary et al., 2017; Rudramurthy et al., 2017).

Herein, the aim of our study is to establish a multiplex assay that can identify *C. auris*, *C. haemulonii*, *C. duobushaemulonii*, and *C. pseudohaemulonii*. Simplicity and affordability of our assay allow the researchers in developing countries to exploit our multiplex PCR assay as a robust screening tool.

## Materials and Methods

### Fungal Isolates

Reference strains for the tetraplex PCR validation comprised *C. auris* (*n =* 138), *C. haemulonii* (*n =* 26), *C. duobushaemulonii* (*n =* 6), and *C. pseudohaemulonii* (*n =* 2) (**Table 1**). Human genomic DNA (Sigma–Aldrich, St. Louis, MO, United States) and 192 other fungal strains ranging from the closest to distant related species were used for specificity testing (**Table 2**). All the samples were obtained from the collection department of Westerdijk Fungal Biodiversity Institute in the form of freeze-dried powder. Subsequently the strains were incubated on Glucose Yeast Extract Peptone Agar plates at 25°C for 48 h, and subsequently pure colonies were confirmed by MALDI-TOF MS (Bruker, Billerica, MA, United States) and LSU sequencing (Vlek et al., 2014; Stielow et al., 2015).

**Table 1:** Reference strains used for tetraplex PCR validation.

Species name	Strain code
*C. auris* (*n =* 138)	CBS 10913; CBS 12372; CBS 12373; CBS 12766; CBS 12767; CBS 12768; CBS 12769; CBS 12770; CBS 12771; CBS 12772; CBS 12773; CBS 12774; CBS 12775; CBS 12776; CBS 12777; CBS-12874; CBS 12875; CBS 12876; CBS 12877; CBS 12878; CBS 12880; CBS 12881; CBS 12882; CBS 12883; CBS 12884; CBS 12887; CBS 12886; Five clinical stains from Oman; 105 clinical strain for Kuwait
*C. haemulonii* (*n =* 26)	CBS 5149; CNM CL4642; CNM CL3458; CNM CL6800; CNM CL7793; CNM CL4640; CNM CL4641; CBS 7801; CBS 5150; CBS 6590; CBS 5468; CBS 7802; CBS 6332; CBS 10968; CBS 10969; CBS 10970; CBS 10971; CBS 10972; CBS 10973; CBS 12439; CBS 6915; CBS 12371; CNM CL7256; CNM CL7462; CNM CL7239T; CNM CL7073
*C. duobushaemulonii* (*n =* 6)	CBS 7800; CBS 7799; CBS 9754; CBS 6915; CBS 7798; CNM CL7829P
*C. pseudohaemulonii* (*n =* 2)	CBS 10004; CBS 12370

**Table 2:** Fungal isolates for specificity test.

Species name	Strain code
*Candida albicans*	CBS 2704; CBS 2691; CBS 2697; CBS 1893; CBS 2712; CBS 5703; CBS 6552; CBS 2689; CBS 2690; CBS 2691; CBS 2695; CBS 2698; CBS 2696; CBS 5137; CBS 2702; CBS 1912; CLF-2; CLF-11; CLF-32; CLF-41; CLF-52; CLF-61; CLF-71; CLF-82; CLF-91; CLF-844; CLF-177; CLF-282; CLF-283; CLF-378; CLF-403; CLF-435; CLF-539; CLF-Llama 1; CLF-Llama 2; CLF-Llama 11
*Candida africana*	CBS 8781
*Candida tropicalis*	CBS 2313; CBS 94; CBS 1920; CLF-6; CLF-15; CLF-26; CLF-36; CLF-45; CLF-56; CLF-65; CLF-75; CLF-86
*Candida parapsilosis*	CBS 11045; CBS 7154; CBS 604; CBS 2197; CBS 2915; CBS 11059; CBS 11920; CBS 8050; CBS 2216; CBS 1954; CBS 2194; CBS 2211; CBS 11043; CBS 11359; CBS 7248; CBS 7156; CBS 10947; CBS 11130; CBS 7157; CBS 8836; CBS 2215; CBS 8181; CBS 12025; CBS 7155; CBS 6318; CBS 2196; CBS 2195; CLF-8; CLF-17; CLF-47; CLF-58; CLF-67; CLF-77; CLF-88; CLF-97
*Candida metapsilosis*	CBS 10907; CBS 2916; CBS 10747; CBS 11127; CBS 2315
*Candida orthopsilosis*	CBS 8825; CBS 9894; CBS 9348; CBS 10741; CBS 10906; CBS 11337; CBS 9347; CBS 10743; CBS 2212; CBS 10744; CBS 11698; CBS 10745; CBS 8548
*Candida glabrata*	CLF-4; CLF-13; CLF-24; CLF-34; CLF-43; CLF-63; CLF-73; CLF-83; CLF-84; CLF-93; CLF-310; CLF-611A; CLF-Llama 3; CLF-Llama 24; CLF- Llama 63
*Candida krusei*	CBS 5147; CLF-30; CLF-40; CLF-60; CLF-69; CLF-79; CLF-99; CLF-611B
*Candida norvegensis*	CBS 6564
*Pichia cactophila*	CBS 6926
*Candida lusitaniae*	CBS 6936; CLF-19
*Candida famata*	CBS 767
*Candida dubliniensis*	CLF-10; CLF-49
*Candida guilliermondii*	CBS 7099
*Candida kefyr*	STA#63
*Candida sake*	CBS 159
*Candida rugosa*	CBS 613
*Candida zeylanoides*	CBS 619
*Yarrowia lipolytica*	CBS 6124
*Pichia fermentans*	CBS 187
*Pichia kluyveri*	CBS 188
*Pichia membranifaciens*	CBS 107
*Pseudozyma thailandica*	CBS 10006
*Kluyveromyces lactis var. lactis*	CBS 683
*Kluyveromyces marxianus*	CBS 712
*Kodamaea ohmeri*	CBS 5367
*Lindnera fabianii*	CBS 5640
*Lindnera jadinii*	CBS 1600
*Lodderomyces elongisporus*	CBS 2605
*Magnusiomyces capitatus*	CBS 162.8
*Metschnikowia pulcherrima*	CBS 5833
*Meyerozyma caribbica*	CBS 9966
*Millerozyma farinosa*	CBS 185
*Ogataea polymorpha*	CBS 4732
*Cryptococcus neoformans*	CBS 8710; CBS 6885
*Cryptococcus deneoformans*	CBS6900; CBS10511
*Cryptococcus gattii*	CBS 7229; CBS 6289
*Cryptococcus bacillisporus*	CBS 6955; CBS 8755
*Cryptococcus deuterogattii*	CBS 10514; CBS 7750
*Cryptococcus tetragattii*	CBS 10101;
*Cryptococcus decagattii*	CBS 11687
*Cryptococcus amylolentus*	CBS 6039
*Rhodotorula mucilaginosa*	CBS 316
*Saccharomyces cerevisiae*	CBS 1171
*Saccharomyces paradoxus*	CBS 432
*Trichosporon asahii*	CBS 2479
*Trichosporon inkin*	CBS 5585
*Debaryomyces nepalensis*	CBS 5921
*Exophiala dermatitidis*	CBS 207.35
*Galactomyces candidus*	CBS 615.84
*Hortaea werneckii*	CBS 107.67
*Hyphopichia burtonii*	CBS 2352; CBS 2532
*Torulaspora globosa*	CBS 764
*Wickerhamomyces anomalus*	CBS 5759
*Wickerhamomyces onychis*	CBS 5587
*Zygosaccharomyces rouxii*	CBS 732
*Schizosaccharomyces pombe*	CBS 356
*Schizosaccharomyces japonicus*	CBS 354
*Aspergillus fumigatus*	CBS 114.55
*Aspergillus flavus*	CBS 107.45
*Aspergillus niger*	CBS 102.12
*Aspergillus terreus*	CBS 106.25
*Aspergillus nidulans*	CBS 100.20
*Fusarium moniliforme*	CBS 130180
*Fusarium solani*	CBS 101427
*Aspergillus versicolor*	CBS 106.57
*Fusarium oxysporum*	CBS 100.97

### DNA Sample Preparation

DNA extraction from pure colonies of yeast cells was performed using the CTAB method as previously described (Gupta et al., 2004). DNA was purified from blood and tissue (kidney) samples using the animal tissue and blood DNA isolation kit (DENAzist Asia, Mashhad, Iran) according to the manufacturer’s instructions.

### Primer Design and PCR Amplification

**FIGURE 1:**
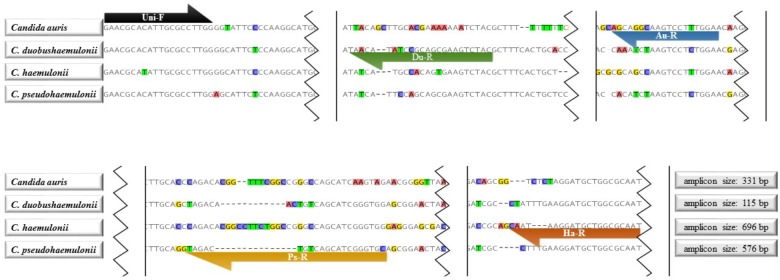
Primer sequences and positions used in the tetraplex PCR. Uni-F: Universal forward primer for *Candida auris*, *C. duobushaemulonii*, *C. haemulonii*, and *C. pseudohaemulonii*; Du-R: *C. duobushaemulonii* specific reverse primer; Au-R: *C. auris* specific reverse primer; Ps-R: *C. pseudohaemulonii* specific reverse primer; Ha-R: *C. haemulonii* specific reverse primer.

The 26s rDNA sequence were obtained from our own in-house database or from NCBI Nucleotide Database^1^ and searched for species-specific and universal regions for primer design. Kordalewska et al. (2017) classified 47 *C. auris* isolates from India, Pakistan, Venezuela, Japan, and South Africa into four clades using Genome-wide SNP-based phylogenetic analyses, which indicated there is high genetic heterogeneity within *C. auris* globally. Considering this and also the large number of new cases after 2016, we designed the primers for *C. auris* by analyzing more isolates from a wider range. For the reverse specific primer for *C. auris*, we analyzed the 26S rDNA sequence of 233 isolates from Kuwait, Japan, Korea, Pakistan, South Africa, Venezuela, India, Malaysia, Israel, United States, and Oman. 137 sequences were done by ourselves and the rest were downloaded from Genbank. By alignment, genetic heterogeneity was easily found in 26S rDNA. Accordingly, a region which is stable within *C. auris*, and specific to other species was chosen for reverse primer. The primer system used in this assay were shown in **Figure 1**, which contained one universal forward primer (Uni-F: 5′-GAACGCACATTGCGCCTTGG-3′) and four species-specific primers (*C. auris*: Au-R, 5′-TCCAAAGGACTTGCCTGCT-3′; *C. duobushaemulonii*: Du-R, 5′-GTAGACTTCGCTGCGGATATGTTA-3′; C. *haemulonii*: Ha-R, 5′-ATTGCGCCAGCATCCTTATTG-3′; *C. pseudohaemulonii*: Ps-R, 5′-GCACCCGATGCTGACAGTCTAC-3′). Primers were synthesized by Integrated DNA Technologies, Coralville, IA, United States.

A total volume of 50 μl PCR mixture containing 5 μl of buffer, 1.5 mM magnesium chloride, 2.5 units of *Taq* polymerase enzyme (BIO-21040, BioLine Company, London, United Kingdom), 0.2 mM dNTP (BIO-39043, BioLine Company, London, United Kingdom), 5 pM Uni-F primer, 5 pM Du-R, 2 pM Ps-R, 3 pM Au-R, and 10 pM Ha-R, 1 μl template DNA or single colony pick (≈1 mm^3^), and 38.3 μl Mili-Q water (Millipore Corporation, Billerica, MA, United States) was used for amplification of DNA from target species.

The PCR was performed using a 2720 thermal cycler (Applied Biosystems, Waltham, MA, United States), and the PCR program consisted of 5 min pre-denaturation at 94°C, followed by 35 cycles of 30 s at 94°C, 30 s at 62°C, 30 s at 72°C and 8 min at 72°C as final extension.

### Validation via Animal Model

When certain infected human samples are not easily available for diagnostic validation, animal model can be an alternative (Bialek et al., 2001). To evaluate its potential use for infected human blood or tissue, animal models infected by the most important pathogens in *Metschnikowiaceae* clade, i.e*., C. auris* and *C. haemulonii*, were developed for diagnostic purpose. All procedures related to animal experiments were approved by the ethics and research committee of Mazandaran University of Medical Sciences, Sari, Iran.

*Candida auris* (CBS 10913, type strain), *C. haemulonii* (CBS 5149, type strains) and *C. albicans* (ATCC 29008, control) were cultured twice at 35°C for 48 h on Sabouraud dextrose agar (SDA, Difco). All isolates were subcultured in brain heart infusion broth and incubated at 37°C with shaking at 150 rpm. Supernatants were carefully removed and washed twice in sterile phosphate buffered saline (PBS). After centrifugation, the cells were washed in PBS and a hemocytometer was used to count the yeast cell. The yeast cell concentration was adjusted to an inoculum size of 5 × 10^5^ yeast/ml. The viable counts of isolates were confirmed by 10-fold serially diluting the cell suspension on SDA plates.

Immunocompetent female ICR (CD-1 specific pathogen- free) mice with a mean weight of 22 g (purchased from Royan Institute, Tehran, Iran) were used in the study. Animals were housed at the accredited Animal Experimentation Facility in standard cages, received sterilized food and were monitored daily (Fakhim et al., 2018). Seven mice were allocated into each of the three mice groups infected with *C. auris*. *C. haemulonii* and *C. albicans*. Infection was induced in the mice for each group with an inoculum of 5 × 10^5^ CFU/mouse in a volume of 0.2 ml into the lateral tail vein. No immunosuppressive scheme was used. Mice were checked daily and euthanized when symptoms of disseminated infection were detected. Any animal that had more than one of the criteria including, decreased activity, hunched posture, torticollis or barrel rolling, inability to eat or drink and hypothermia was humanly euthanized by intracardiac puncture under general anesthesia (Conti et al., 2014). Tissue sections were also stained with hematoxylin and eosin (H&E) for microscopic examination. Tissue (kidney) and blood samples were recovered under aseptic conditions. Tissue samples were homogenized in sterile saline and 100 μl of homogenate was cultured on SDA at 35°C to confirm *Candida* infection and determine the colony forming units (CFU) were determine (Fakhim et al., 2018). All yeast cells cultures were identified by our tetraplex PCR. In addition, 100 μl of each EDTA-blood sample was cultured on SDA plates. The rest of the homogenate and blood samples were used for tetraplex PCR test.

### Application in Clinical Setting

A single-center prospective clinical screening for *C. auris*, *C. duobushaemulonii*, *C. haemulonii* and *C. pseudohaemulonii* was performed during 2017.05.01 to 2017.11.01 in Shanghai Changzheng Hospital, China. The newly developed multiplex PCR system worked as a supplementary tool to BD BACTEC^TM^ FX40 Instrument (Becton, Dickinson and Company, Franklin Lakes, NJ, United States), CHROMagar^TM^ Candida (CHROMagar Microbiology, Paris, France) and microscopic examination to finding infection cases by the targeted species. In order to further test the specificity of our assay using more clinical isolates, we used a wide range of sample types for screening, including blood, sputum, feces, bronchoalveolar lavage fluid and oral swab. We test all the clinical yeast isolates that are identified by CHROMagar^TM^ Candida as non-*albicans Candida* species. As soon as the clinical culture is obtained by conventional diagnostic methods, single colony (≈1 mm^3^) is tested by this multiplex PCR system. DNA extraction and ITS sequencing are done as well to confirm the accuracy of the assay in the clinical settings as described before (Stielow et al., 2015).

Two hundred and fifty five clinical non-*albicans Candida* isolates (identified by CHROMagar^TM^ Candida) from Mazandaran University of Medical Sciences, Iran, were used as a retrospective screening sample in this study. The isolates were collected from blood, sputum, feces, oral swab and vaginal samples during 2016.1–2017.6. Multiplex PCR were performed, and MALDI-TOF and LSU sequencing were done as well to confirm the accuracy of the assay in the clinical settings (Vlek et al., 2014; Stielow et al., 2015).

All the tests were approved by the ethics and research committees of Shanghai Changzheng Hospital and Mazandaran University of Medical Sciences.

## Results

### Analytical Validation

**FIGURE 2:**
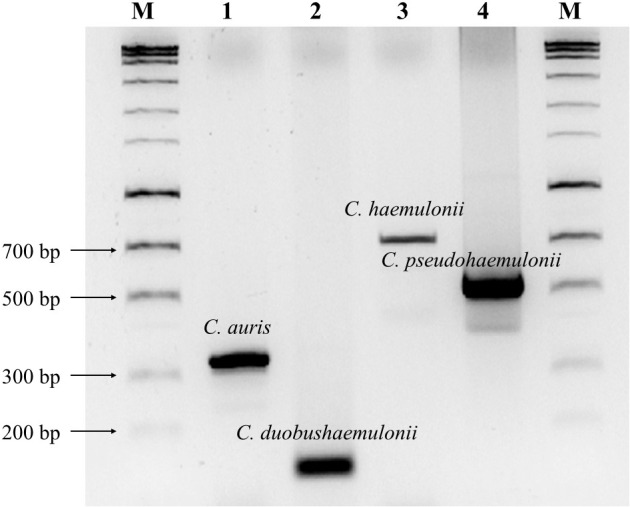
Agarose gel electrophoresis (2% agarose) of PCR amplicons. Lanes 1–4 are *C. auris* (CBS 10913 type, 331 bp), *C. duobushaemulonii* (CBS 7798 type, 115 bp), *C. haemulonii* (CBS 5149 type, 696 bp) and *C. pseudohaemulonii* (CBS 10004 type, 576 bp), respectively. Lane M, HyperLadder^TM^ 100 bp (Bioline, London, United Kingdom).

**Figure 2** showed that four amplicons exhibited major bands that could be differentiated from each other. The gel figure for all the strains of *C. auris* and its relatives tested in this study was shown in **Supplementary Figure S1**. Sequencing results of PCR amplicons were 100% matched with the targeted species. Human genomic DNA and 192 other fungal species were tested, and no cross-reactivity was found (100% specificity).

### Animal Model Testing

**FIGURE 3:**
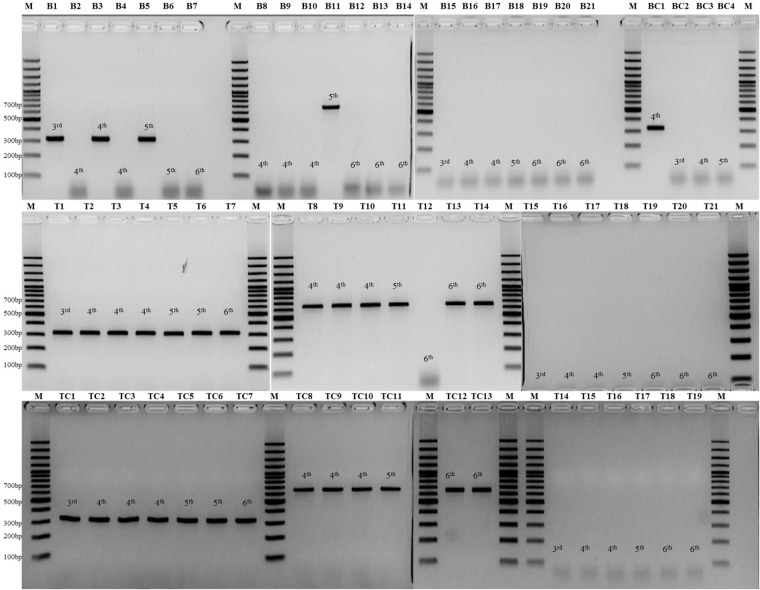
Lanes B1–B7 are PCR results of blood sample from seven mice infected with *C. auris*; Lanes B8-B14 are PCR results of blood sample from seven mice infected with *C. haemulonii*; Lanes B15-B21 are PCR results of blood sample from seven mice infected with *C. albicans*; Lane BC1–4 are PCR results of four cultured strains from blood samples of all the 21 mice; Lanes T1–T7 are PCR results of kidney tissue sample from seven mice infected with *C. auris*; Lanes T8–T14 are PCR results of kidney tissue sample from seven mice infected with *C. haemulonii*; Lanes T15–T21 are PCR results of kidney tissue sample from seven mice infected with *C. albicans*; Lane TC1–7 are PCR results of seven cultured strains from kidney samples of mice infected with *C. auris*; Lane TC8–13 are PCR results of six cultured strains from kidney samples of mice infected with *C. haemulonii*; Lane TC14–19 are PCR results of six cultured strains from kidney samples of mice infected with *C. albicans*; Lane M, GeneRuler 100 bp Plus DNA Ladder (Thermo Fisher, Waltham, MA, United States).

**Figure 3** showed the detailed information of the animal model testing.

Application of the tetraplex PCR in blood showed a higher overall positive rate (4 of 14, 28.6%) than blood culture positive rate (1 of 14, 7.1%) in animal models of *C. auris* and *C. haemulonii*. Specifically, three *C. auris* (3/7, on the 3rd, 4th, and 5th day post-infection (PI)) and one *C. haemulonii* (1/7, on the 5th day, PI) infected mice showed positive PCR results from blood, without cross-reactivity in *C. albicans* group (0/7). Culture results were positive only in one blood samples of *C. auris* (1/7, on the 4th day, PI), negative in all *C. haemulonii* infected mice (0/7), and positive in three *C. albicans* infected mice (3/7, on the 3rd, 4th, and 5th day PI), with CFUs ranging from 1 to 6 colonies in 100 μl.

The overall positive PCR rate of mice kidney samples was 92.8% (13/14) among *C. auris* and *C. haemulonii*, without cross-reactivity in *C. albicans* group (0/7). Among the kidney samples from 21 mice examined by culture, 19 were positive due to *C. auris* (7/7), *haemulonii* (6/7), and *C. albicans* (6/7) with CFUs ranging from 8.3 × 10 to 4.3 × 10^4^ colonies in 100 μl (mean 3.5 × 10^3^ colonies).

### Clinical Validation

Applying our assay in a prospective (566 clinical isolates) and a retrospective cohorts (255 clinical isolates), revealed one *C. haemulonii* isolate (S49AF, accession number: KY112738) from Iran and one *C. haemulonii* isolate (SCZ90793, accession number: MG637448), one *C. duobushaemulonii* isolate (SCZ91445, accession number: MG963993) and one *C. pseudohaemulonii* isolate (SCZ90800, accession number: MG242063) from China. Specifically, the Chinese *C. pseudohaemulonii* was isolated from sputum of a 65-year-old male who suffered from lower esophageal resection (Shanghai, China). To our knowledge this the first isolate of C. *pseudohaemulonii* from China. Whereas the Iranian *C. haemulonii* isolate was recovered from toenail sample of a 36-year-old diabetic male in 2016 (Shiraz, Iran), and also was the first *C. haemulonii* isolate from Iran.

## Discussion

Achieving a timely and accurate diagnosis of infections caused by *C. auris* and its phylogenetic relatives is of great difficulty in clinical settings (Mizusawa et al., 2017). Nearly all the *C. auris* isolates were misdiagnosed as *C. haemulonii*, *C. sake*, *C. famata*, *Rhodotorula glutinis*, *Saccharomyces cerevisiae* by clinical commercial methods such as Vitek-2 YST ID system, API-20C AUX, AuxaColor 2, BD Phoenix, and MicroScan (Chowdhary et al., 2017). Studies also showed that *C. pseudohaemulonii* failed to be distinguished from *C. haemulonii* by these methods (Kim et al., 2009). All the identifications from the biochemical assays should be confirmed by sequencing. The Centers for Disease Control of America speculated that the unavailability of appropriate diagnostic method underestimate its prevalence in many countries (Sarma and Upadhyay, 2017), which includes medical resource-limited regions. Hence, we developed a novel end-point tetraplex PCR system in a low-cost, rapid and accurate format. This novel assay has been validated by a large number of CBS/clinical isolates, diagnostic animal models and clinical application. We hope this assay can help to understand the global clinical impact of *C. auris* and its relatives, especially in the under-served and poor regions.

Our new end-point tetraplex PCR was designed to identify and differentiate the most medically important and related species to *C. auris*. Recently, two real-time PCR based assays for *C. auris* were developed (Kordalewska et al., 2017; Leach et al., 2017). Compared with the real-time PCR method, our end-point PCR is non-automated and labor-intensive. The electrophoresis step of our assay normally required 45 min, which is more time-consuming than real-time PCRs. Additionally, since the nature of end-point PCR, our assay is unable to quantify the fungal load of host. However, the advantages of our assay over theirs are also obvious.

Kordalewska et al. (2017) established the first SYBR Green real-time PCR system for *C. auris*, *C. duobushaemulonii*, *C. haemulonii*, and *C. lusitaniae*; While Leach et al. (2017) developed a probe-based singleplex real-time PCR only for *C. auris*. Considering the high misdiagnostic rate between *C. auris, C. haemulonii*, *C. duobushaemulonii*, and *C. pseudohaemulonii*, and higher resistance of *C. pseudohaemulonii* to AMB than *C. auris, C. haemulonii, C. duobushaemulonii*, and *C. lusitaniae* (Kim et al., 2009; Shin et al., 2012), we used a multiplex strategy for this assay and included *C. pseudohaemulonii* instead of *C. lusitaniae.* Among the selected species, *C. haemulonii* initially was known as a multi-antifungal resistant pathogen since the first case report in 1984, and it can cause outbreaks and infect immunocompetent individuals (Loosova et al., 2001; Khan et al., 2007). Subsequently, *C. haemulonii* was recognized as a complex species and reclassified as *C. haemulonii, C. duobushaemulonii*, and *C. haemulonii var. vulnera* (Cendejas-Bueno et al., 2012). *C. auris* and *C. pseudohaemulonii* were described in 2009 and 2006, respectively. Both of them were multi-antifungal resistant pathogens and the latter is spreading across the world at an accelerated pace (Sugita et al., 2006; Chowdhary et al., 2017). Correct species-level identification for the four species is needed due to the clinical importance and heterogeneity in terms of taxonomy, spreading prevalence and antifungal susceptibility profile.

Our multiplex PCR system is the first assay for *C. auris* that has been validated by animal model testing. The overall PCR positive rates of mice blood and tissue were 28.6 and 92.9%, respectively, which showed slightly higher performance than culture based identification. And PCR/culture from tissue samples showed higher value than those from blood for diagnosing infections caused by these pathogens. Moreover, our new assay is much faster than culture based identification. PCR directly from tissue/blood only requires 3 h from sample preparation to result read-out; while culture based identification is more time-consuming (normally 1–3 days).

Our new assay is also featured by its low cost and potential to be used in medical resource-limited settings. Due to the poor economic condition, lack of basic medical facilities and the relatively poor population health, the developing countries were reported to have a much higher risk of infections than the developed ones (Pittet et al., 2008). Among the 17 countries reported with *C. auris* prevalence, seven are the developing ones (Oman, Venezuela, Colombia, South Africa, Kenya, Pakistan, and India). Considering this, our new assay was established to help to extend the global knowledge of the epidemiology of these important and emerging pathogens, especially for medical resource-limited countries. Compared with MALDI-TOF and real-time PCR cycler, end-point PCR cycler used in this assay is more portable and much cheaper ($130–$4,000 per cycler) (Wong et al., 2015). For clinical validation, we used isolates from two clinical settings that can represent different situations of medical care in developing countries (Shanghai, China and Sari, Iran). In Shanghai, specific diagnostic tools such as MALDI-TOF and sequencing are available to most hospitals. However, since China is a developing country, there are still a large number of patients that cannot afford these tests. While in Iran, to our knowledge, there is no MALDI-TOF and sequencing machine. There was only one epidemiology study related to *C. haemulonii* and *C. duobushaemulonii* in China and no epidemiology data is available in Iran (Hou et al., 2016). Hence, we hold the prevalence of *C. auris* and its close related relatives are underestimated due to the lack of low-cost and accurate assays in both countries. By using this assay, we successfully found the first clinical isolates of *C. haemulonii* in Iran and first clinical isolates of *C. pseudohaemulonii* in China. However, no *C. auris* isolate was found in both cohorts.

## Conclusion

The novel end-point tetraplex PCR for *C. auris* and its phylogenetic relatives is rapid and 100% specific. Clinical validation and animal model testing further proved its clinical performance. This assay only requires the simple devices of end-point PCR cycler and electrophoresis instrument, showing its availability and affordability to most of the developing countries, and can contribute to a better understanding of the clinical occurrence of *C. auris* and its relatives.

## Ethics Statement

This study was carried out in accordance with the recommendations of Guide for the Care and Use of Laboratory Animals, committee of the Update of the Guide of the Care and Use of Laboratory Animals. The protocol was approved by the Ethics and Research Committee of Mazandaran University of Medical Sciences, Sari, Iran (No. 2321).

## Author Contributions

AA and WF participated in primer design, PCR optimization, data collection, and drafted the manuscript. WP, FH, and TB participated in designing this study and revising the manuscript. HB and AV participated in animal model testing and collecting Iranian clinical isolates. WJ participated in clinical validation in China. All authors contributed to the writing of the final manuscript.

## Conflict of Interest Statement

The authors declare that the research was conducted in the absence of any commercial or financial relationships that could be construed as a potential conflict of interest.

## Footnotes

1 https://www.ncbi.nlm.nih.gov/nuccore/

## Supplementary Material

FIGURE S1 Gel figure for all the strains of *C. auris* and its relatives tested in this study.
