# Supplementary figures and images for: Low-Cost Tetraplex PCR for the Global Spreading Multi-Drug Resistant Fungus, Candida auris and Its Phylogenetic Relatives
Source: Front Microbiol. 2018 May 29;9:1119. doi: 10.3389/fmicb.2018.01119 (PMC5987591; doi:10.3389/fmicb.2018.01119)

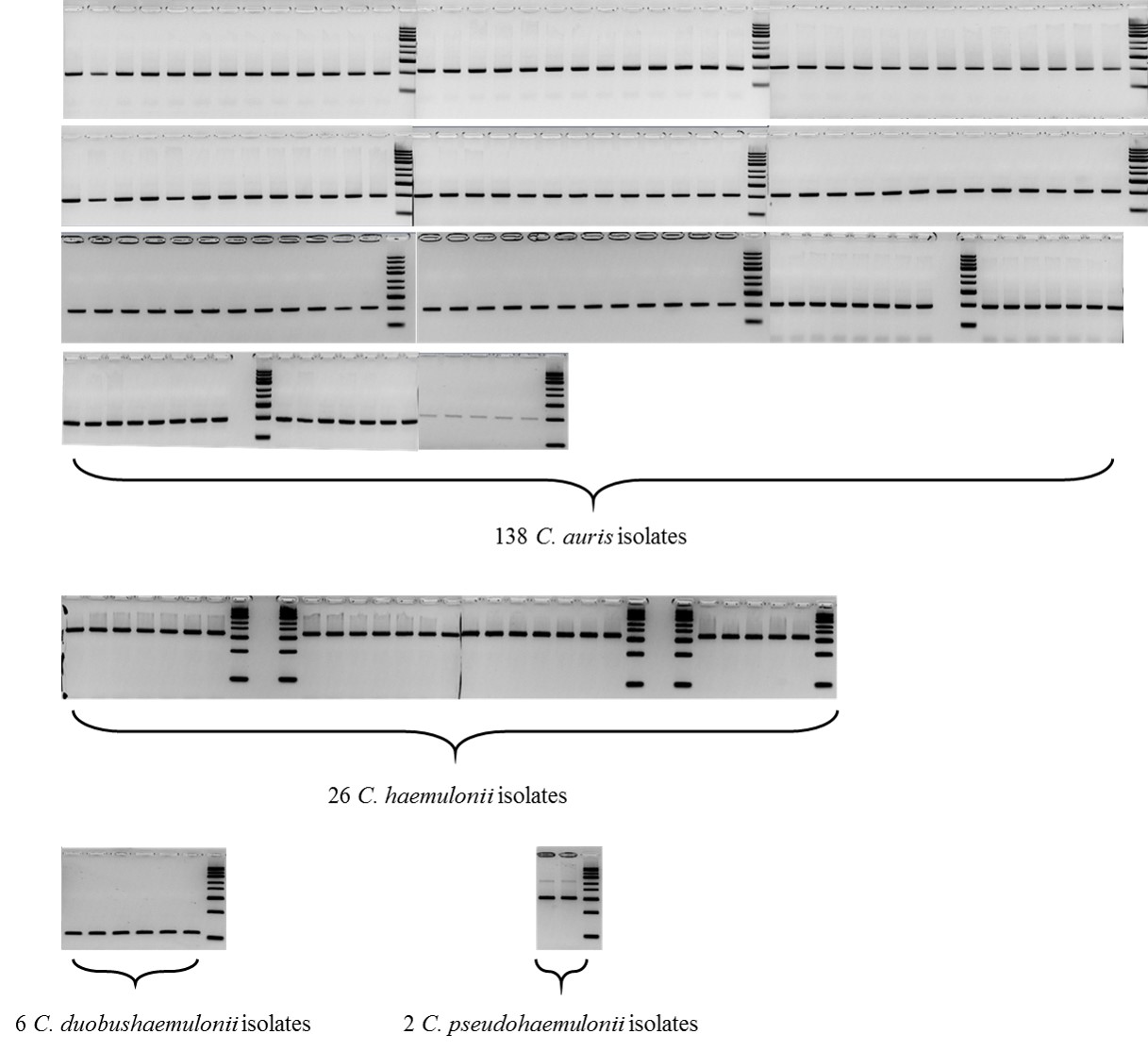

Supplement: FIGURE S1 — Gel figure for all the strains of C. auris and its relatives tested in this study. [file Image_1.JPEG]
